# Atherosclerosis is an inflammatory disease: treat it as such!

**DOI:** 10.1007/s00392-024-02470-x

**Published:** 2024-05-28

**Authors:** Markus Therre, Philip Wenzel, Bernhard Haring

**Affiliations:** 1 Department of Internal Medicine III, Cardiology, Angiology and Intensive Care Medicine, University Hospital Saarland, Saarland University, Kirrberger Str. 100, Homburg, 66421 Saar, Germany; 2 Department of Cardiology, University Medical Center of the Johannes Gutenberg University Mainz and German Center for Cardiovascular Research – Partner Site Rhine-Main, Mainz, Germany; 3 Department of Epidemiology and Population Health, Albert Einstein College of Medicine, Bronx, NY USA

Sirs:

All patients with atherosclerotic disease should receive lipid-lowering therapy for prevention of cardiovascular events, with multiple approved and effective pharmacological therapies currently available [1]. Unfortunately, a substantial proportion of patients suffer from recurrent adverse events despite optimal management of traditional cardiovascular risk factors including low-density lipoprotein (LDL) cholesterol [2]. Among underlying explanatory factors, the residual inflammatory risk hypothesis has been postulated, referring to the increased risk of CV events caused by vascular inflammation [3]. In fact, inflammation, as measured by high-sensitivity CRP (hsCRP) levels, has recently shown to be even stronger than residual LDL cholesterol risk for the prediction of cardiovascular events in certain patients under statin therapy [4]. Several adjunct anti-inflammatory agents were demonstrated to improve cardiovascular outcomes in high-risk individuals [5–7]. To date, low-dose colchicine is the only approved and guideline-recommended anti-inflammatory agent for the prevention of recurrent cardiovascular events in patients receiving statins [8]. However, enthusiasm about the effectiveness of anti-inflammatory therapies in general is hampered by the fact that all-cause mortality could not be reduced by any of the drugs investigated. Thus, patient populations that benefit most from recent and future anti-inflammatory therapies have yet to be characterized.

In this issue of *Clinical Research in Cardiology*, Bay et al. present results on clinical outcomes in patients undergoing percutaneous coronary intervention (PCI) for chronic coronary syndrome (CCS) according to inflammatory markers and number of affected vascular beds by atherosclerosis using a large and contemporary single-center registry [Reference Clin Res Cardiology]. Elevated hsCRP values were defined as > 3 mg/l, and polyvascular atherosclerotic disease (polyVD) was defined as additional diagnosis of cerebrovascular or peripheral artery disease. Mean hsCRP values were significantly higher in patients suffering from polyVD than those without. Patients with both elevated hsCRP and polyVD had the highest rates of major adverse cardiovascular events. Consequently, the authors identified an independent association between hsCRP and major adverse cardiovascular events among the polyVD group, suggesting a significant residual inflammatory risk in these patients [Reference Clin Res Cardiology]. Given the reasonable prevalence of patients with CCS and polyVD, these results are relevant for interventional as well as general cardiologists.

The time for addressing residual inflammatory risk in clinical routine has already arrived, with colchicine as effective anti-inflammatory agent being available [9]. Additionally, the cardiometabolic drugs sodium-glucose cotransporter-2 inhibitors and glucagon-like peptide-1 receptor agonists exert pleiotropic atheroprotective effects and are recommended to a large extent for patients with CCS. The well-documented protective effects of these aforementioned substances are not yet fully understood and might be at least partially explained through anti-inflammatory mechanisms, as they have been shown to lower hsCRP levels [10, 11]. In patients with statin intolerance, lipid-lowering bempedoic acid significantly reduced hsCRP levels [12]. Novel anti-inflammatory agents are currently undergoing clinical trials and might provide further treatment opportunities in future [13]. This will be especially relevant for multimorbid patients with polyVD or chronic kidney disease (CKD), where few medical therapies are effective or not feasible. The currently ongoing ZEUS trial (NCT05021835, [14]) investigates the interleukin-6 inhibitor ziltivekimab in patients with significant CKD, a population at high risk for recurrent cardiovascular events.

Despite recent advances in anti-inflammatory therapy, questions remain and additional research on underlying mechanisms—especially in polyVD and CKD patients—is needed. Do patients with cerebrovascular or peripheral artery disease without affection of the coronary arteries also exhibit significant inflammatory risk? With coronary artery disease being a heterogeneous entity, what is the best time for the initiation of anti-inflammatory treatment? How do modern cardiometabolic medical therapies affect inflammatory pathways and will their anti-inflammatory effect amplify when combined with novel anti-inflammatory agents? And, lastly, are drugs that target cytokine formation and/or signaling really the most promising intervention, or should we seek for more tailored, spatially and temporally restricted, individualized and cell based therapies? Answering these questions will sharpen our understanding and the definition of target populations for currently available as well as future anti-inflammatory therapies.

Atherosclerosis is an inflammatory disease. As such, lipid-lowering and anti-inflammatory agents should not be considered contradicting but synergistic therapies [9]. The contribution by Bay et al. helps to define target populations who benefit most from anti-inflammatory therapies [Reference Clin Res Cardiology]. This important additional piece of evidence should raise our awareness to evaluate every patient in our daily practice for his or her residual inflammatory risk [8].
